# Loss of SH3GL2 promotes the migration and invasion behaviours of glioblastoma cells through activating the STAT3/MMP2 signalling

**DOI:** 10.1111/jcmm.13184

**Published:** 2017-05-04

**Authors:** Yufu Zhu, Xiang Zhang, Lei Wang, Zhe Ji, Manyi Xie, Xinyu Zhou, Zhiyi Liu, Hengliang Shi, Rutong Yu

**Affiliations:** ^1^ Institute of Nervous System Diseases Xuzhou Medical University Xuzhou China; ^2^ Brain Hospital Affiliated Hospital of Xuzhou Medical University Xuzhou China; ^3^ The Graduate School Xuzhou Medical University Xuzhou China; ^4^ Department of General Surgery Affiliated Hospital of Xuzhou Medical University Xuzhou Jiangsu China

**Keywords:** SH3GL2, STAT3, MMP2, glioma

## Abstract

SH3GL2 (Src homology 3 (SH3) domain GRB2‐like 2) is mainly expressed in the central nervous system and regarded as a tumour suppressor in human glioma. However, the molecular mechanism of the SH3GL2 protein involved in malignant behaviours of human glioma has not been elucidated. In this study, we tried to investigate the role of SH3GL2 in glioma cell migration and invasion and explore its underlined molecular mechanism. Firstly, we discovered that the protein level of SH3GL2 was widely decreased in the human glioma patients, especially in high‐grade glioma tissues. Then, we determined the role of SH3GL2 in migration and invasion of glioma cells upon SH3GL2 knocking down and overexpressing. It was showed that knocking down of SH3GL2 promoted the migration and invasion of glioma cells, whereas overexpression of SH3GL2 inhibited them. Further study on molecular mechanism disclosed that silencing of SH3GL2 obviously activated the STAT3 (signal transducer and activator of transcription 3) signalling thereby promoting the expression and secretion of MMP2. On the contrary, overexpression of SH3GL2 had opposite effect. Taken together, the above results suggest that SH3GL2 suppresses migration and invasion behaviours of glioma cells through negatively regulating STAT3/MMP2 signalling and that loss of SH3GL2 may intensify the STAT3/MMP2 signalling thereby contributing to the migration and invasion of glioma cells.

## Introduction

Glioma is the most universal type of primary intracranial tumours 1,which accounts for more than 70% of all brain tumours 2. It displays a persistent malignant progression featured by widespread infiltration throughout the brain 3. The prognosis of glioma patients is still very poor. The median survival time is approximately 15 month, and fewer than 3% of glioblastoma patients can survive for 5 years after diagnosis 2. Therefore, studying the mechanisms of glioma and prolonging the survival time of patients become urgent and significant matters we are facing.

The SH3GL2 gene was cloned by Sparks *et al*. in 1996 and mapped to chromosome 9p22 4. *SH3GL2* gene has nine exons and encodes a 352 amino acid protein also named Endophilin‐1 4, 5, 6, 7, 8. Endophilin‐1 is a multifunctional protein, and most of its functions associate with synaptic vesicle endocytosis and regulating intracellular signalling 9, 10, 11, 12, 13. Recent studies indicate that SH3GL2 functions as a tumour suppressor that particularly distributes in the central nervous system 4, 5. Increasing evidence shows that SH3GL2 is less expressed in a variety of carcinomas, including breast carcinoma 14, non‐small cell lung cancer 15, laryngeal carcinoma 16, urothelial carcinoma 17, and head and neck squamous cell carcinoma 18. In glioma, SH3GL2 is decreasingly expressed and correlated with the incidence of glioblastoma 19. In addition, miRNA‐330 has been found to play a role in promoting human glioblastoma by inhibiting SH3GL2 gene 20. These data indicate that SH3GL2 may serve as a tumour suppressor in human glioblastoma; however, the potential molecular mechanism involved still needs to be clarified.

STAT3 is abnormally activated in glioblastoma and has been considered as a valuable therapeutic target in this disease and numerous other human cancers 21. It has been proved that loss of SH3GL2 is associated with aggressive disease and promotes oncogenic activities including up‐regulation of STAT3 17. As a transcription factor and activator, STAT3 links to metastatic progression of multiple different cancer types, including lung, skin, liver, ovarian, kidney and colon cancer 22. STAT3 activation may be a critical event in the formation of metastasis. It involves in tumour metastases through a variety of pathways in these cancers 22, 23, 24. However, the mechanism that STAT3 promotes glioma malignant behaviours remains poorly understood.

Recent studies have suggested that STAT3 enhances MMP2 expression by directly interacting with the promoter of MMP2 *via* a STAT3‐binding element 23, which is critical for cell invasion in many cancers, including glioblastoma 25, 26. Therefore, we speculate that STAT3/MMP2 signalling may be involved in SH3GL2 mediated migration and invasion of glioma cells.

In this study, we firstly examined the protein expression of SH3GL2 in glioma patients and glioma cell lines by Western blotting and immunohistochemistry. Then, the role of SH3GL2 in the migration and invasion glioma cells was investigated through silencing or overexpressing approaches. Finally, we studied the effect of SH3GL2 on STAT3/MMP2 signalling.

## Materials and methods

### Antibodies

SH3GL2 antibody was purchased from Abcam (Cambridge, UK). Antibodies specific for MMP2, STAT3, p‐STAT3, FLAG and β‐actin were purchased from Cell Signaling Technology (Danvers, MA, USA); SU9516 ([Z]‐1,3‐dihydro‐3‐[1H‐imidazol‐4‐ylmethylene]‐5‐methoxy‐2H‐indol‐2‐one), a potent and selective CDK2 inhibitor, was purchased from Tocris Bioscience (Bristol, UK).

### Tissue samples

Thirty‐three specimens of human glioma tissues (surgical resection) and nine specimens of non‐tumorous brain tissues (internal decompression in cerebral trauma) were collected at the Affiliated Hospital of Xuzhou Medical University (Xuzhou, China). All glioma specimens had confirmed pathological diagnosis and were classified according to the World Health Organization (WHO) criteria. Written informed consent was obtained from each patient, and the study was approved by the Research Ethics Committee of Xuzhou Medical University.

### Cell culture

Glioma cell lines U251, U87, A172, U118, C6 and human embryonic kidney cell line HEK293T were bought from Shanghai Cell bank, Type Culture Collection Committee, Chinese Academy of Sciences. The cells were cultured in Dulbecco's modified Eagle's medium (DMEM) (Invitrogen, Carlsbad, CA, USA) supplemented with 10% foetal bovine serum (TransGen, Beijing, China) and grown in a humidified incubator with 5% CO_2_ at 37℃.

### Constructs and production of the lentivirus

For silencing of SH3GL2, three short hairpin RNA (shRNA) sequences were designed as follows:

SH3GL2 #1‐F: 5′‐GATCGGATGAAGAGCTTCGTCAATTCAAGAGATTGACGAAGCTCTTCATCCTTTTTTG‐3′,
SH3GL2 #1‐R: 5′‐AATTCAAAAAAGGATGAAGAGCTTCGTCAATCTCTTGAATTGACGAAGCTCTTCATCC‐3′;
SH3GL2 #2‐F: 5′‐GATCGGAGATGGATATTGAACAATTCAAGAGATTGTTCAATATCCATCTCCTTTTTTG‐3′,
SH3GL2 #2‐R: 5′‐AATTCAAAAAAGGAGATGGATATTGAACAATCTCTTGAATTGTTCAATATCCATCTCC‐3′;
SH3GL2 #3‐F: 5′‐GATCGCCTAGAAGGGAATATCAATTCAAGAGATTGATATTCCCTTCTAGGCTTTTTTG‐3′,
H3GL2 #3‐R:
5′‐AATTCAAAAAAGCCTAGAAGGGAATATCAATCTCTTGAATTGATATTCCCTTCTAGGC‐3′;
Control‐F,5′‐GATCTTCTCCGAACGTGTCACGTTTCAAGAGAACGTGACACGTTCGGAGAATTTTTTG‐3′,
Control‐R, 5′‐AATTCAAAAAATTCTCCGAACGTGTCACGTTCTCTTGAAACGTGACACGTTCGGAGAA‐3′.

The shRNA and control oligomers were annealed and then subcloned into the pLV‐shRNA plasmid by the *BamH* I and *EcoR* I cloning sites. Cell transfection was carried out with PolyJet (SignaGen, Gaithersburg, MD, USA) as described in the manufacturer's protocol. The lentiviruses were produced in HEK293T cells by cotransfecting the corresponding plasmids with the helper plasmids. For overexpression of SH3GL2, the SH3GL2 construct was generated by the human SH3GL2 cDNA was cloned into the vector p3 × FLAG‐CMV‐14 (3*FLAG) at the *Hind* III and *BamH* I restriction sites.

### Establishment of the stable cell lines

The construction of stable cell lines was carried out as we previously described 27, 28. For stable silencing of SH3GL2, the U251 cells were infected by control and SH3GL2 #1‐3 viruses, respectively. Forty‐eight hours after infection, the cells were continuously cultured in the medium supplemented with 2.5 μg/ml puromycin (Sigma, St. Louis, MO, USA). The surviving cells were developed into cell lines stably expressing control or SH3GL2 #1‐3.

### Wound healing assay

The cells were seeded in six‐well plates under normal conditions for 24 hrs. Then, the scratches were developed in the middle of the wells with a pipette tip. Thereafter, the cells were cultured for an additional 48 hrs in the presence of SU9516. Photographs were harvested with an inverted microscope (IX71, Olympus Tokyo, Japan) at the designated time‐points. The number of cells crossing the wound was normalized to the control.

### Transwell invasion and migration assays

Transwell assays were performed with a polycarbonate filter membrane with a diameter of 6.5 mm and pore size of 8 μm (Invitrogen) according to the manufacturer's protocol. To assess invasion ability, the filters were pre‐coated with 10 μg of Matrigel (BD). The cell suspension (1 × 10^5^) in serum‐free culture media was added into the inserts, and each insert was placed in the lower chamber filled with culture media containing 10% foetal bovine serum as a chemoattractant. After 24 hrs of incubation at 37°C, the non‐invasive cells were removed from the upper chamber by wiping with cotton‐tipped swabs. Then, the filters were fixed with methanol for 15 min. and stained with a 0.1% crystal violet solution for 10 min. Five fields of adherent cells in each well were randomly photographed with an inverted microscope and counted. The same experimental design was used for migration experiments except that filters were not pre‐coated with Matrigel.

### RNA isolation, cDNA synthesis and RT‐PCR

RNA was extracted from stable lines or transfected cells, and the cDNA was synthesized using the M‐MLV Reverse Transcriptase (Invitrogen) according to the manufacturer's instruction. Quantitative RT‐PCR was carried out by an ABI7300 real‐time PCR instrument (Applied Biosystems, Carlsbad, CA, USA) using SYBR Green. Primers for the amplification of SH3GL2, MMP2 and GAPDH were as follows:

SH3GL2‐F: 5‐AAGCAGTTCCATAAAGCCACT‐3,
SH3GL2‐R: 5‐TCCTGGCCACGGATTTTTGA‐3;
MMP2‐F: 5‐CAGGATCATTGGCTACACACC‐3,
MMP2‐R: 5‐CCATACTTCACACGGACCACT‐3;
GAPDH‐F: TGGAGTCCACTGGCGTCTTC,
GAPDH‐R: CATTGCTGATGATCTTGAGGCT.

### Gelatin zymography assay

MMP2 secretion into the conditioned media was determined by gelatin zymography. The cells were cultured in serum‐free media for 48 hrs, and the conditioned media was harvested, centrifuged and resuspended in SDS loading buffer without β‐mercaptoethanol. In each condition, equal amounts of protein were loaded on a 10% SDS‐PAGE, supplementing 1 mg/ml gelatin as substrate. Zymograms were isolated in a Tris/glycine SDS running buffer under non‐denaturing conditions. After electrophoresis, SDS remnants were removed by washing gels with 2.5% Triton X‐100. Zymograms were subsequently reacted with a MMP substrate buffer (50 mM Tris‐HCl, 10 mM CaCl2, 150 mM Nacl pH 7.5) for 16 hrs at 37°C. After incubation, the gels were stained with 0.25% Coomassie brilliant blue R250 for 3 hrs at room temperature and then destained by 30% methanol and 10% acetic acid until the bands of lysis become clear. The proteolytic activity was visualized as the development of clear bands under a blue background.

### Immunohistochemical Analysis

Immunohistochemical (IHC) staining was carried out according to the protocol supplied by the S‐P immunohistochemistry kit (Zhongshan Goldenbridge Biotech Co., Beijing, China). The sections were fixed with 4% paraformaldehyde and blocked with 10% goat serum. Then, the sections were incubated with SH3GL2 antibody (1:50 dilution, PBS as blank), followed by Biotin‐conjugated anti‐rabbit IgG and HRP‐conjugated streptavidin. Subsequently, the reactions were developed by 3′‐diaminobenzidine (DAB) chromogenic reagent (Zhongshan Goldenbridge Biotech Co.). Then, the sections were counterstained with haematoxylin and dehydrated by incubation in gradient concentrations of alcohol, followed by 100% xylene. Finally, the coverslips were mounted onto the slides with neutral gum. The photographs were collected under an Olympus IX‐71 microscope (Olympus).

### Western blotting

At the designated time, the cells were lysed and equal amounts of protein were isolated on a 10% SDS‐PAGE and then transferred to 0.45‐μm pore size PVDF membrane (Millipore, Billerica, MA, USA). After blocking with 5% non‐fat milk, the membrane was probed with primary antibodies (SH3GL2, MMP2, p‐STAT3, STAT3, FLAG and β‐actin) at 4°C overnight. On the following day, membranes were incubated in a horseradish peroxidase‐labelled goat anti‐rabbit/mouse IgG and detected by enhanced chemiluminescence detection system (Thermo Fisher, Waltham, MA, USA). Band densities were quantified by ImageJ Software (Wayne Rasband, National Institutes of Health, MD). The relative amount of each protein was determined by normalizing the densitometry value of interest to that of the loading control.

### Statistical analysis

The results were representative of experiments repeated at least three times, and quantitative data were expressed as means ± S.E.M. Statistical analyses were carried out using the SPSS Version 13.0 (SPSS Inc, Chicago, IL). Differences in multiple groups were determined by a one‐way analysis of variance (ANOVA) followed by post hoc test. Comparison between two groups was performed by Student's *t*‐test. *P* values <0.05 were considered statistically significant (*P < 0.05).

## Results

### SH3GL2 protein is down‐regulated in human glioma tissues and glioma cells

**Figure 1:**
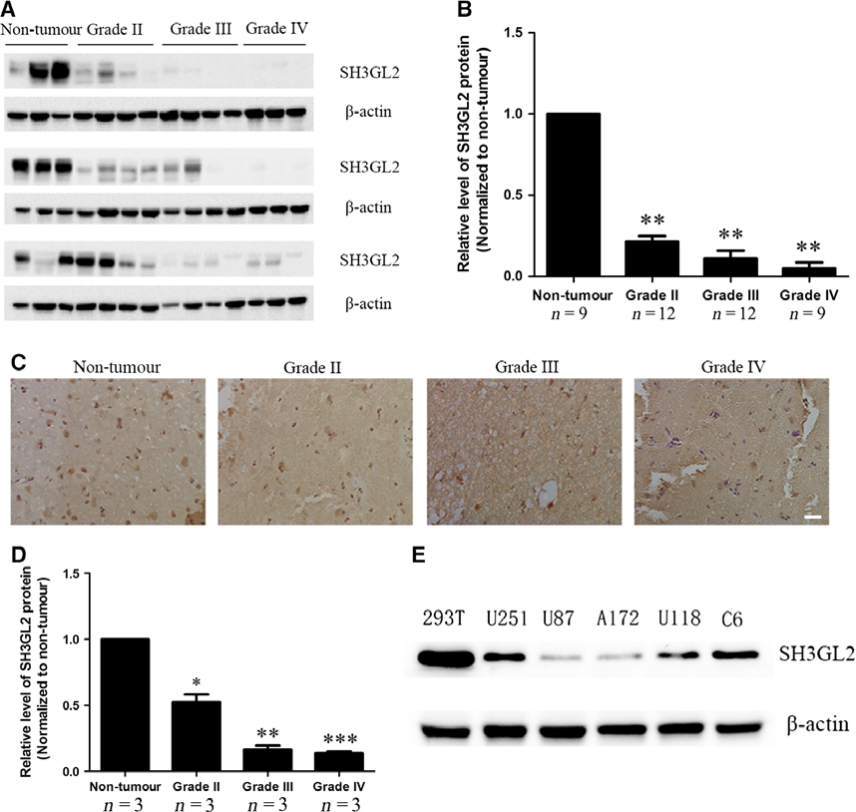
Expression of SH3GL2 in human glioma patients and glioma cell lines. (**A**) Total proteins isolated from non‐tumorous brain tissue and glioma tissues were analysed by Western blotting for assessment of SH3GL2. (**B**) Statistical chart showed the expression level of SH3GL2 in non‐tumorous brain tissue and the different grades of glioma tissues. The ratios indicate the levels of SH3GL2 to β‐actin levels with respect to each sample. (**C**) Representative images of SH3GL2 from non‐tumorous brain tissues and human gliomas (grades II‐IV) determined by immunohistochemistry, scale bars: 20 μm. (**D**) Statistical chart showed the expression level of SH3GL2 in non‐tumorous brain tissue and the different grades of glioma tissues. (**E**) Expression of SH3GL2 in non‐tumorous cell line (293T) and glioma cell lines (U251, U87, A172, U118, C6). *P < 0.05, **P < 0.01 and ***P < 0.001.

To investigate the possible role of SH3GL2 in the development of human glioma, total lysates were extracted from 33 specimens of human glioma tissues (twelve grade II, twelve grade III and nine grade IV) and nine specimens of human non‐tumorous brain tissues, and the SH3GL2 protein level was evaluated by Western blotting. As shown in Figure 1A and B, the expression level of SH3GL2 was dramatically decreased in glioma tissues, especially in high‐grade glioma tissues (grades II‐IV). Immunohistochemical analysis also showed that expression of SH3GL2 was significantly decreased in glioma tissues compared to non‐tumorous tissues (Fig. 1C and D). Furthermore, we examined the SH3GL2 expression level in non‐tumorous cell line (293T) and a variety of glioma cell lines (U251, U87, A172, U118 and C6). The results showed that the expression of SH3GL2 in glioma cell lines was dramatically decreased compared to the non‐tumorous cell line 293T, especially in U87 cell line with a higher degree of malignancy (Fig. 1E). These results reveal that the protein expression of SH3GL2 is down‐regulated in human glioma, which provides us the preliminary evidence that SH3GL2 may play a role in the progression of human glioma.

### Down‐regulation of SH3GL2 promotes glioma cell migration and invasion

**Figure 2:**
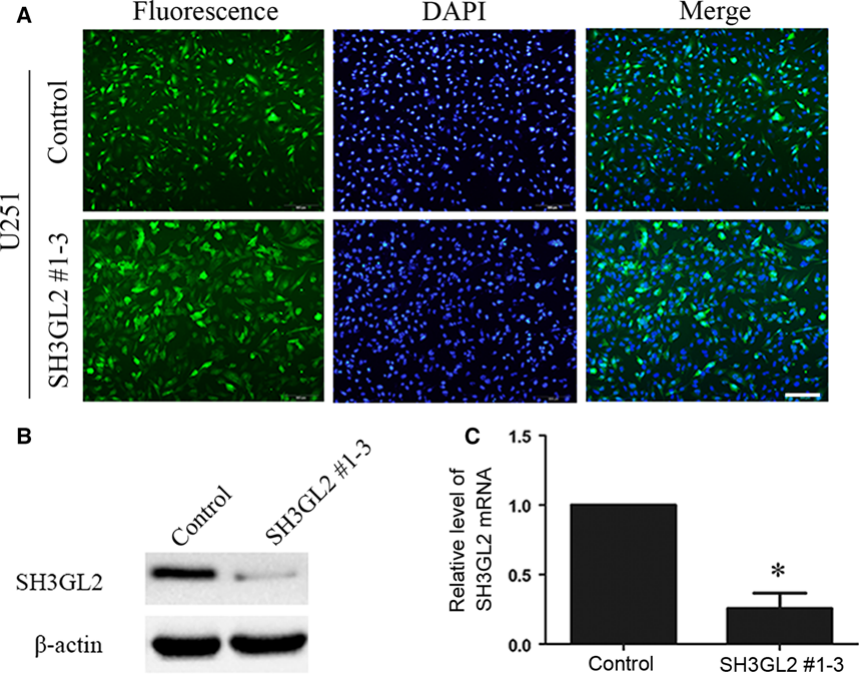
Silencing of SH3GL2 in U251 cells. (**A**) The GFP photographs showed the stable cell line that silencing of SH3GL2 in U251 cells, scale bars: 200 μm. (**B**) The silencing efficiency of SH3GL2 #1‐3 was examined by Western blotting. (**C**) The silencing efficiency of SH3GL2 #1‐3 was verified by RT‐PCR. *P < 0.05.

**Figure 3:**
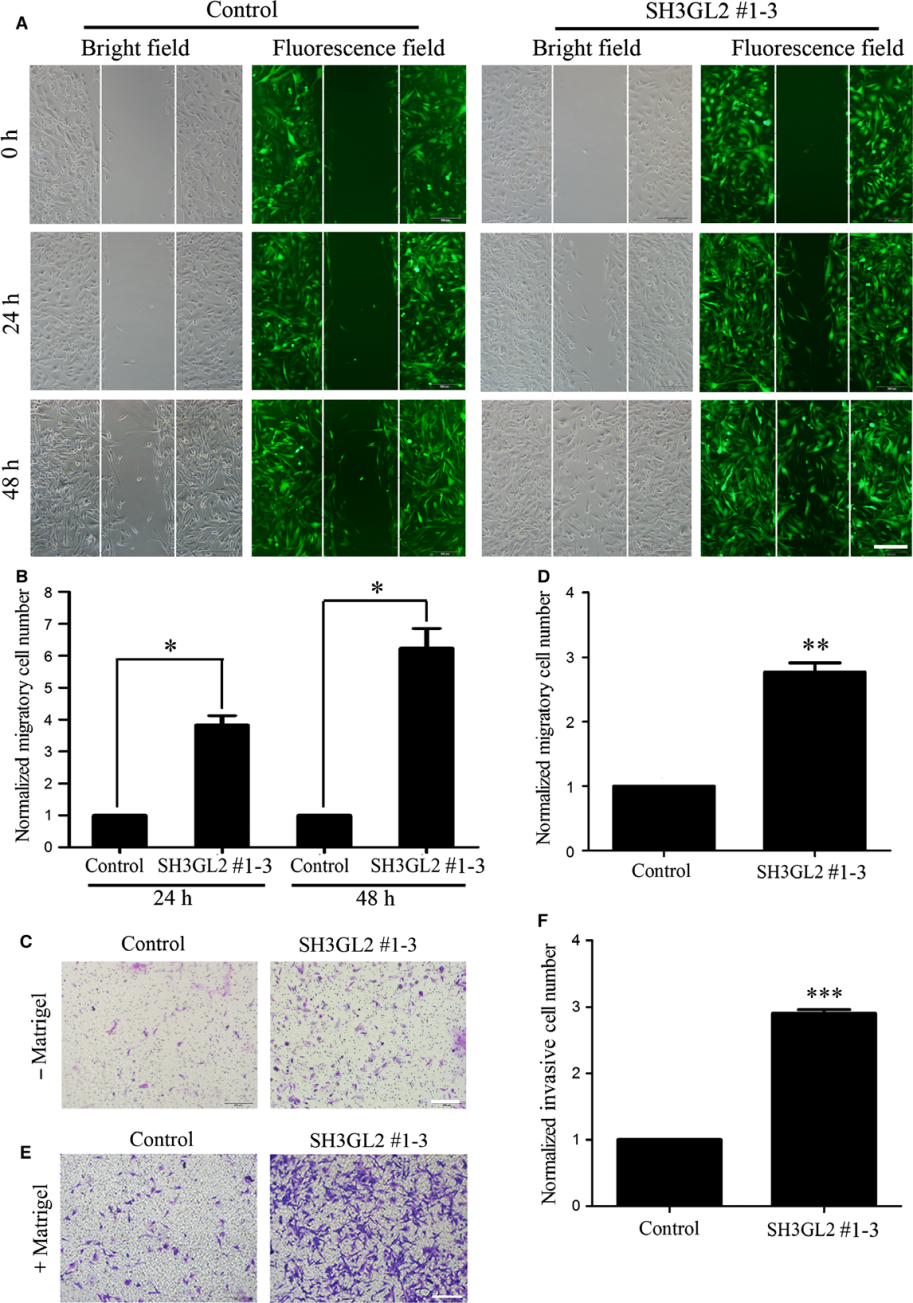
Down‐regulation of SH3GL2 promotes glioma cell migration and invasion. (**A**) Wound healing assay with control and SH3GL2 #1‐3 expressing U251 cells, scale bars: 500 μm. (**B**) Statistical analysis showed that inhibition of SH3GL2 increased glioma cell migration compared with control. (**C**) Transwell migration assay with control and SH3GL2 #1‐3 expressing U251 cells, scale bars: 200 μm. (**D**) Statistical analysis showed that inhibition of SH3GL2 increased glioma cell migration compared with control. (**E**) Transwell invasion assay with control and SH3GL2 #1‐3 expressing U251 cells, scale bars: 200 μm. (**F**) Statistical analysis showed that inhibition of SH3GL2 increased glioma cell invasion compared with control. *P < 0.05, **P < 0.01, ***P < 0.001.

As high‐grade glioma usually has a strong ability of invasion, we focused on exploring the possible roles of SH3GL2 in the migration and invasion of glioma cells. For this purpose, we employed knocking down approach in U251 cell line that has a high level of SH3GL2 and used overexpressing strategy in U87 cell line that has a low level of SH3GL2. Firstly, we down‐regulated SH3GL2 expression using its specific shRNA and observed the effects on cell migration and invasion. For silencing of SH3GL2, three shRNA targets (SH3GL2 #1, SH3GL2 #2 and SH3GL2 #3) were screened for their efficacy in suppressing SH3GL2 expression, and a negative non‐targeting shRNA was used as a control. We found that using three shRNA alone did not have an ideal silencing efficiency; however, three shRNA mixtures could successfully knock down the expression of SH3GL2 (Data not shown). The off‐target effect of the shRNAs was excluded by testing their effect on human and mouse SH3GL2 (Fig. S1). Thereafter, the SH3GL2 #1, SH3GL2 #2 and SH3GL2 #3 were packaged into the lentivirus to co‐infect the U251 cells to develop the stable cell lines with loss of SH3GL2 (Fig. 2A). The silencing efficiency was confirmed by Western blotting and quantitative RT‐PCR experiments (Fig. 2B and C). Next, we detected whether the cell migration and invasion were affected by knocking down of SH3GL2 in the stable U251 cell lines. The wound healing assay indicated that, compared with the control group, the number of migratory cells was approximately increased by 270% at 24 hrs and 500% at 48 hrs, respectively (Fig. 3A and B). The transwell migration assay (− Matrigel) obtained similar results with the wound healing assay (Fig. 3C and D). The transwell invasion assay (+ Matrigel) displayed that the number of invasive cells was approximately increased by 195%, compared with the control group (Fig. 3E and F). These results suggest that SH3GL2 is involved in the migration and invasion of human glioma cells and down‐regulation of SH3GL2 promotes the migration and invasion of glioma cells.

### Overexpression of SH3GL2 inhibits glioma cell migration and invasion

**Figure 4:**
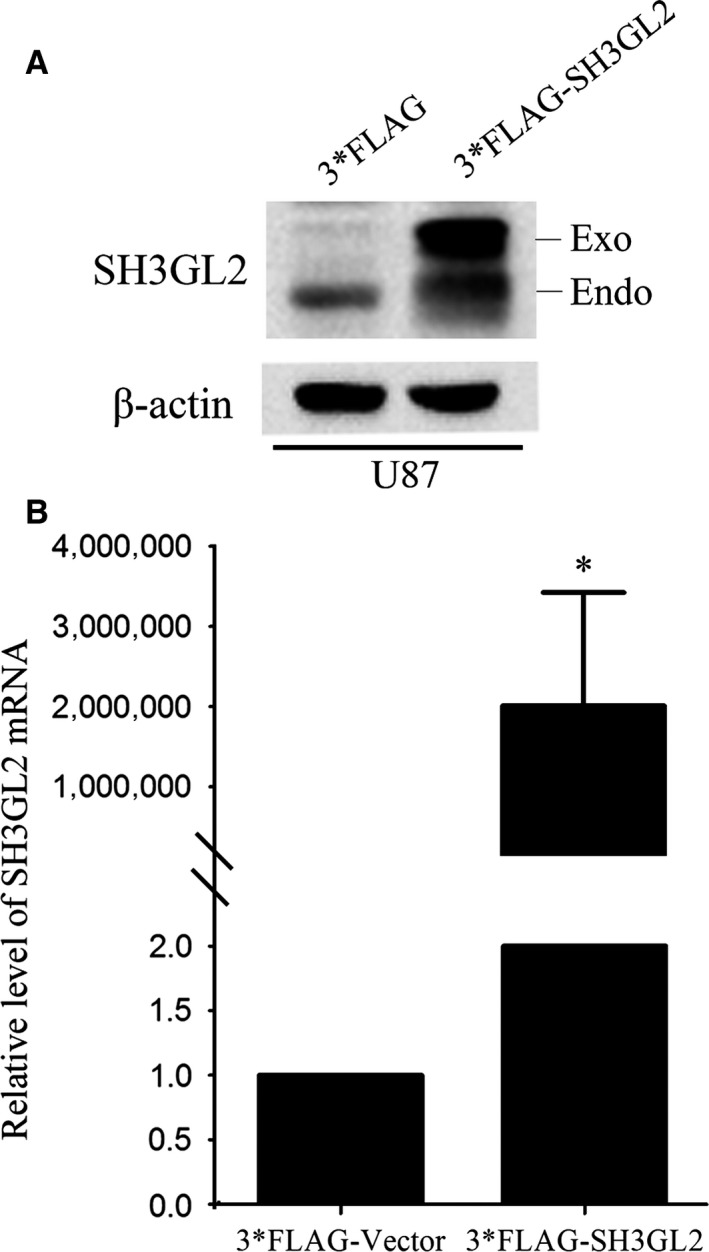
Overexpressing of SH3GL2 in U87 cells. (**A**) The overexpressing efficiency of 3*FLAG‐SH3GL2 was examined by Western blotting. (**B**) The overexpressing efficiency of 3*FLAG‐SH3GL2 was examined by RT‐PCR. *P < 0.05.

**Figure 5:**
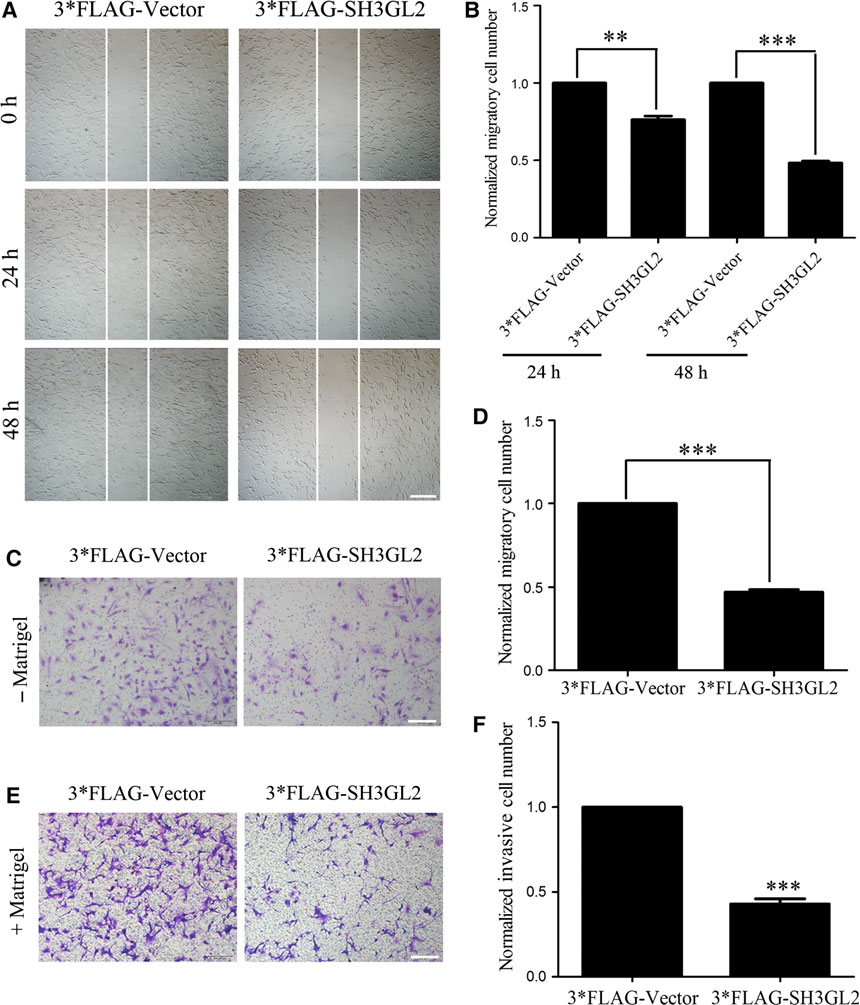
Overexpression of SH3GL2 inhibits glioma cell migration and invasion. (**A**) Wound healing assay with 3*FLAG‐Vector or 3*FLAG‐SH3GL2 transfected U87 cells, scale bars: 500 μm. (**B**) Statistical analysis showed that overexpression of SH3GL2 inhibited glioma cell migration compared with the control. (**C**) Transwell migration assay with 3*FLAG‐Vector or 3*FLAG‐SH3GL2 transfected U87 cells, scale bars: 200 μm. (**D**) Statistical analysis showed that overexpression of SH3GL2 inhibited glioma cell migration compared with the control. (**E**) Transwell invasion assay with 3*FLAG‐Vector or 3*FLAG‐SH3GL2 transfected U87 cells, scale bars: 200 μm. (**F**) Statistical analysis showed that overexpression of SH3GL2 inhibited glioma cell invasion compared with the control. **P < 0.01, ***P < 0.001.

To further determine the role of SH3GL2 in glioma cell migration and invasion, we then transiently transfected 3*FLAG‐tagged SH3GL2 cDNA into U87 cells to achieve the gain‐of function. The expression efficiency of SH3GL2 was confirmed by Western blotting and quantitative RT‐PCR experiments (Fig. 4A and B). Then, we asked whether the cell migration and invasion were aggravated upon SH3GL2 overexpression. Therefore, 24 hrs after transfection, the cells were used to perform the wound healing and transwell assay. The wound healing assay revealed that, compared with the 3*FLAG group, the number of migratory cells of 3*FLAG‐SH3GL2 group was approximately reduced by 25% and 55% at 24 and 48 hrs after transfection, respectively (Fig. 5A and B). The transwell migration assay (‐ Matrigel) obtained similar results with the wound healing assay (Fig. 5C and D). The transwell invasion assay (+ Matrigel) indicated that the number of invasive cells was approximately reduced by 60% (Fig. 5E and F) compared with the corresponding control. These results further suggest that SH3GL2 is involved in the migration and invasion of human glioma cells and overexpression of SH3GL2 inhibits the glioma cell migration and invasion.

### SH3GL2 negatively regulates STAT3/MMP2 signalling pathway

**Figure 6:**
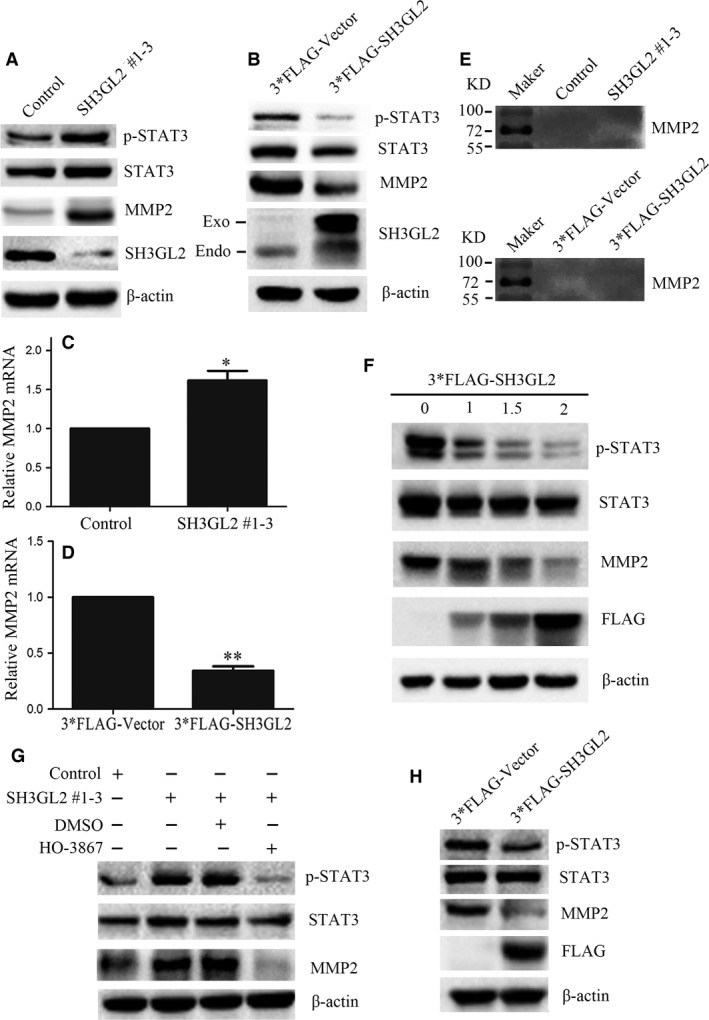
SH3GL2 negatively regulates STAT3/MMP2 signalling pathway. (**A**) Representative blots showed the levels of STAT3, p‐STAT3 and MMP2 in control or SH3GL2 silenced U251 cells. (**B**) Representative blots showed the levels of STAT3, p‐STAT3 and MMP2 in 3*FLAG‐Vector or 3*FLAG‐SH3GL2 overexpressed U87 cells. (**C**) RT‐PCR showed that silencing of SH3GL2 significantly up‐regulated the mRNA of MMP2. (**D**) RT‐PCR showed that overexpression of SH3GL2 significantly down‐regulated the mRNA of MMP2. (**E**) Gelatin zymogram assay showed that silencing of SH3GL2 increased the secretion of MMP2, while overexpressing of SH3GL2 decreased it. (**F**) Western blotting results showed that the expression of MMP2 and p‐STAT3 were gradually decreased along with the increase in 3*FLAG‐SH3GL2 amounts. (**G**) Western blotting results showed that silencing of SH3GL2 induced up‐regulation of p‐STAT3 and MMP2 could be significantly blocked by the STAT3 inhibitor, HO‐3867. (**H**) Western blotting results showed that SH3GL2‐STAT3/MMP2 signalling cascade also existed in PTEN intact C6 cells line. *P < 0.05 and **P < 0.01.

Recent studies have indicated that silencing of SH3GL2 activated STAT3 signalling, while overexpressing of SH3GL2 inhibited it 15, 17. It was also verified that activation of STAT3 could up‐regulate the transcription of matrix metalloproteinase 2 (MMP2) through direct binding to its promoter. Based on these knowledge,we speculated that SH3GL2 might negatively regulate STAT3 which would affect the expression of MMP2 in human glioma cells. To address our question, we firstly detected the protein levels of STAT3 and p‐STAT3 in SH3GL2 silenced U251 cells and SH3GL2 overexpressed U87 cells by Western blotting. As shown in Figure 6A, silencing of SH3GL2 in U251 cells significantly up‐regulated the level of p‐STAT3, while overexpression of SH3GL2 in U87 cells dramatically down‐regulated it (Fig. 6B). Furthermore, we examined the mRNA and protein levels of MMP2 by quantitative RT‐PCR and Western blotting assays, respectively. It was showed that the levels of MMP2 mRNA and protein were significantly increased upon silencing of SH3GL2 (Fig. 6C), but decreased upon overexpression of SH3GL2 (Fig. 6D), which was consistent with the secretion of MMP2 demonstrated from gelatin zymography assay (Fig. 6E). In order to further investigate the above question, we used different amounts of 3*FLAG‐SH3GL2 (0 μg, 1 μg, 1.5 μg, 2.0 μg) to transiently transfect U87 cells. Western blotting results indicated that the expressions of MMP2 and p‐STAT3 were gradiently decreased along with the increase in 3*FLAG‐SH3GL2 amounts (Fig. 6F). Most importantly, to confirm SH3GL2‐STAT3/MMP2 are on the same pathway, STAT3 inhibitor HO‐3867 was used to perform the rescue experiment. It was showed that silencing of SH3GL2 in U251 cells significantly up‐regulated the levels of p‐STAT3 and MMP2, which could be significantly blocked by the STAT3 inhibitor, HO‐3867 (Fig. 6G). Besides, the U251 and U87 cell lines we used are all PTEN mutated 29, 30. To demonstrate the STAT3/MMP2 signalling cascade is PTEN independent, we reproduced the experiment in a PTEN intact C6 cell line 31, 32, 33. It was found that overexpression of SH3GL2 in C6 cells also could significantly down‐regulate the protein levels of p‐STAT3 and MMP2 (Fig. 6H). Taken together, these results illustrate that SH3GL2 may play an important role in inhibiting glioma cell migration and invasion by suppressing STAT3/MMP2 signalling pathway.

## Discussion

SH3GL2 is mainly distributed in central nervous system, particularly enriched in the presynaptic ganglion 4. It is a multifunctional gene, except for its endocytic functions, the non‐endocytic functions of SH3GL2 may play a crucial role in the malignant progression of cancer. Previous studies have shown that SH3GL2 is less expressed and acts as a tumour suppressor in several cancer types including laryngeal cancer, breast cancer and so on 14. It is reported that SH3GL2 expression is obviously lower in glioblastoma tissues comparing with normal brain tissues, indicating that the SH3GL2 may act as a tumour suppressor in human glioblastoma 19. However, the molecular mechanisms of the SH3GL2 protein involved in human glioblastoma are not completed clear, which need to be further clarified. In this study, we tried to elaborate the molecular mechanism of SH3GL2 in glioma cell migration and invasion. The obtained results proved that knocking down of SH3GL2 markedly aggravated the migration and invasion of glioma cells by activating the STAT3 signalling thereby promoting the expression and secretion of MMP2. In contrast, overexpression of SH3GL2 had opposite effect. These results show that SH3GL2 suppresses migration and invasion behaviours of glioma cells through negatively regulating STAT3/MMP2 signalling.

Inhibition of specific tumour suppressor genes and activation of specific oncogenes have been demonstrated to be important for the occurrence and malignant progression of glioblastoma 34, 35, 36, 37. STAT3 has been considered as a valuable therapeutic target in glioma because (*i*) multiple signalling pathways changed in glioma converge to STAT3 and (*ii*) it involves in multiple characteristics of glioma aggressiveness, *via* regulation of genes especially implicated in cell proliferation, growth, apoptosis, migration, invasion and neoangiogenesis 38. The sustained activation of STAT3 in glioma is therefore due to an abnormal signal from upstream regulators. This includes, on the one hand, any gain‐of function mutation or up‐regulation in activation of an upstream activator, and, on the other hand, any loss‐of function mutation or down‐regulation in activation of an upstream suppressor. SH3GL2 has been reported to regulate endocytosis of receptor tyrosine kinases implicated in oncogenesis, such as the epidermal growth factor receptor (EGFR) 15, 17. In this study, we discovered that the protein expression of SH3GL2 was widely suppressed in glioma patients, especially in high‐grade glioma patients. This will cause the aberrant activation of STAT3/MMP2 signalling, which facilitated the migration and invasion of glioma cells. Therefore, loss of SH3GL2 may be an critical factor in the occurrence and malignant progression of glioma.

In conclusion, we have presented the first evidence linking SH3GL2 to malignant behaviours of human glioblastoma through STAT3/MMP2 pathway. These findings will provide some basis for further investigation of SH3GL2‐mediated signalling pathway and for evaluation of the prognostic utility of SH3GL2 status in patients with malignant glioma. However, more deeply studies are needed to clarify the precise mechanisms of SH3GL2‐mediated migration and SH3GL2‐mediated invasion in human malignant glioma.

## Conflict of interest

The authors declare that they have no conflict of interest.

## Supporting information


**Fig. S1** The off‐target effect of the shRNAs was excluded by testing their effect on human and mouse SH3GL2.
