# Supplementary figures and images for: Loss of SH3GL2 promotes the migration and invasion behaviours of glioblastoma cells through activating the STAT3/MMP2 signalling
Source: J Cell Mol Med. 2017 May 4;21(11):2685–94. doi: 10.1111/jcmm.13184 (PMC5661104; doi:10.1111/jcmm.13184)

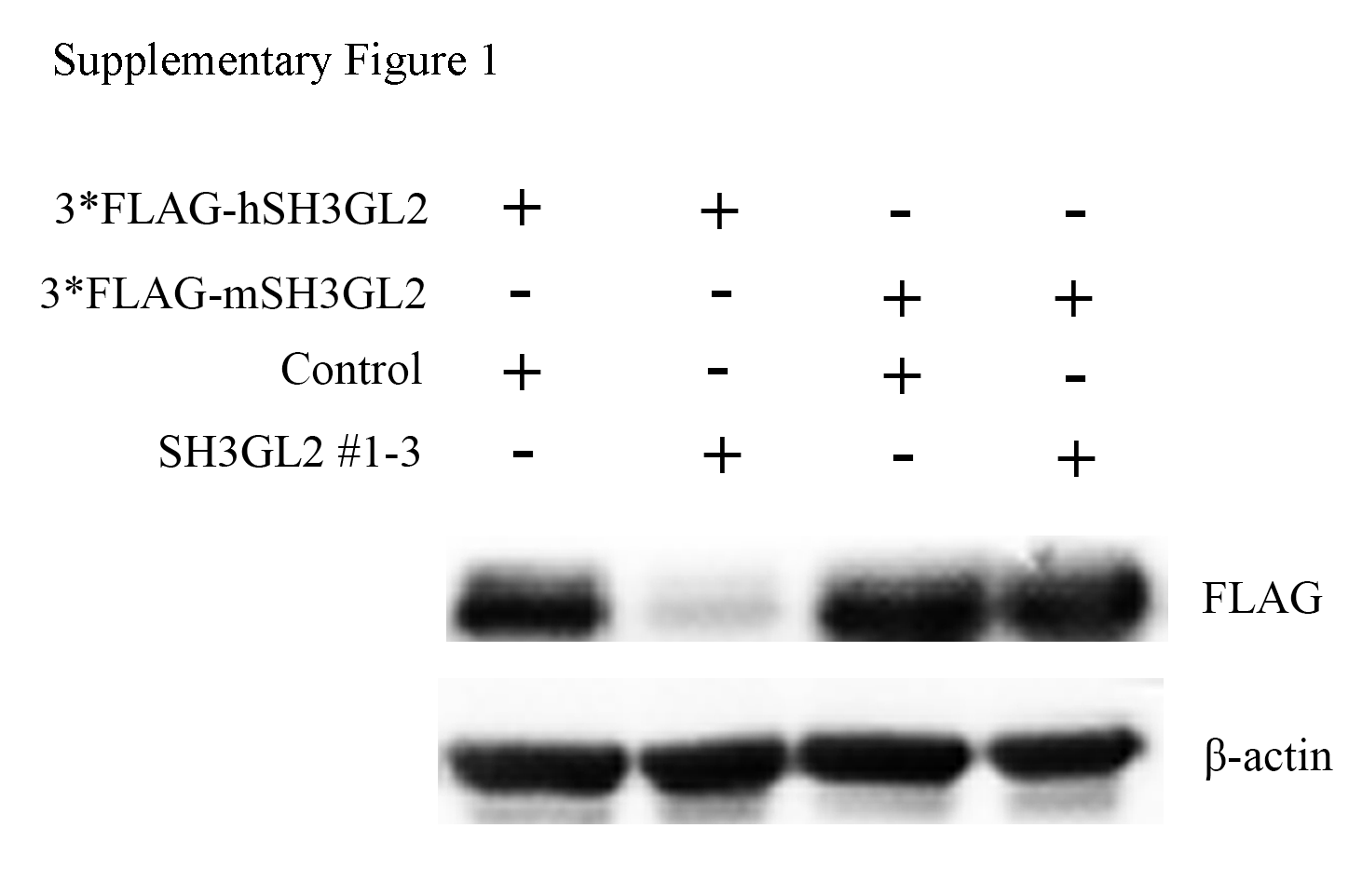

Supplement: Supplementary file 1 — Fig. S1 The off‐target effect of the shRNAs was excluded by testing their effect on human and mouse SH3GL2. [file JCMM-21-2685-s001.tif]
